# EM and XRM Connectomics Imaging and Experimental Metadata Standards

**Published:** 2025-12-29

**Authors:** Miguel E. Wimbish, Nicole K. Guittari, Victoria A. Rose, Jorge L. Rivera, Patricia K. Rivlin, Mark A. Hinton, Jordan K. Matelsky, Nicole E. Stock, Brock A. Wester, Erik C. Johnson, William R. Gray-Roncal

**Affiliations:** Research and Exploratory Development Department, Johns Hopkins University Applied Physics Laboratory, Laurel, MD, USA

**Keywords:** Connectomics, Neuroanatomy, Electron Microscopy, X-ray Microtomagraphy, Data Standards, FAIR Data

## Abstract

High resolution volumetric neuroimaging datasets from electron microscopy (EM) and x-ray micro and holographic-nano tomography (XRM/XHN) are being generated at an increasing rate and by a growing number of research teams. These datasets are derived from an increasing number of species, in an increasing number of brain regions, and with an increasing number of techniques. Each of these large-scale datasets, often surpassing petascale levels, is typically accompanied by a unique and varied set of metadata. These datasets can be used to derive connectomes, or neuron-synapse level connectivity diagrams, to investigate the fundamental organization of neural circuitry, neuronal development, and neurodegenerative disease. Standardization is essential to facilitate comparative connectomics analysis and enhance data utilization. Although the neuroinformatics community has successfully established and adopted data standards for many modalities, this effort has not yet encompassed EM and XRM/ XHN connectomics data. This lack of standardization isolates these datasets, hindering their integration and comparison with other research performed in the field. Towards this end, our team formed a working group consisting of community stakeholders to develop Image and Experimental Metadata Standards for EM and XRM/XHN data to ensure the scientific impact and further motivate the generation and sharing of these data. This document addresses version 1.1 of these standards, aiming to support metadata services and future software designs for community collaboration. Standards for derived annotations are described in a companion document. Standards definitions are available on a community github page. We hope these standards will enable comparative analysis, improve interoperability between connectomics software tools, and continue to be refined and improved by the neuroinformatics community.

## 1 Introduction

Neuroimaging datasets are rapidly evolving in scale, which may unlock critical insights into neuronal circuit organization and neurodegenerative disease including connectopathies. Supported by large-scale investments, such as the from the National Institutes of Health (NIH) Brain Research Through Advancing Innovative Neurotechnologies (BRAIN) Initiative [1], this growth in scale of neuroimaging is not a niche development as a growing number of research teams and multi-institution teams are generating groundbreaking datasets.

In particular, there have been major advances in EM and XRM/XNH connectomics dataset generation, which enable the creation of increasingly large connectomes (wiring diagrams) with individual neuron and synapse resolution. Recent efforts have included large scale volumes from multiple species and brain regions, with increasing spatial extent. This includes imaging of C. elegans [2, 3], Mus musculus cortex [4, 5, 6, 7, 8], Drosophila [9, 10, 11], and Homo sapiens [12]. Individual image volumes are reaching the scale of hundreds of terabytes or even petabytes of data. New datasets include co-registration to additional modalities [8] as well as imaging of multiple individuals at different developmental time points [3]. In addition to these emerging datasets, there is continued development in imaging techniques, processing algorithms, data storage solutions, and analysis techniques [13, 14, 11, 15, 16, 17]. This proliferation of datasets and techniques hold tremendous promise large-scale, cross-dataset discovery, but also presents considerable challenges.

This growth in neuroimaging is driving the need for sophisticated data management and metadata creation strategies to support the production and dissemination of complex datasets [18, 19]. As a result, the field is seeing a significant increase in collaborative neuroinformatics efforts, that are aimed at effectively storing, processing, and sharing vast and intricate datasets. These platforms include, DANDI [20], BrainLife [21], BossDB [13], BrainCircuits.io [16], OpenNeuro [22], NEMAR [23], BIL [24], NEMO [25], and DABI [26]. These large-scale neuroinformatics efforts span a range of modalities, including neurophysiology, genomics, transcriptomics, and functional and structural neuroimaging. These datasets are most impactful when they can be accessed and integrated across datasets and even modalities. This has been recently demonstrated by the BRAIN Initiative Cell Consensus Network atlas [27] data ecosystem [28], which fused data from across archives to create a comprehensive understanding of cellular organization in mammalian cortex. This collaboration among scientific consortia demonstrates the real-world benefits of Findable, Accessible, Interoperable and Reusable (FAIR) principles for data management [18]. These emerging large-scale datasets are challenging to reproduce and reuse, thus a standardized framework is necessary to enable researchers to evaluate data across these datasets [29]. This also applies to emerging EM and XRM/XNH datasets, particularly as these datasets increase in size and complexity.

Given the recent growth in EM and XRM/XNH connectomics datasets, there is now a presenting need for similar neuroinformatics efforts and standardization for connectomics data. To address the needs of the EM and XRM/XNH connectomics community, tools for data storage, visualization and analysis such as BossDB [13], DVID [17], webKnossos [30], BrainCircuits.io [16], cloud-volume [31], Neuroglancer [32], neuPrint [33], and CAVE [14] are being developed and adopted by EM and biology communities [34]. However, there has not yet been a comprehensive effort to standardize EM and XRM/XNH connectomics datasets and provide metadata guidelines for these tools.

While data standards have been proposed for neurophysiology (Neurodata Without Borders, [20]), neuroimaging (BIDS, [35]), and 3D microscopy [36], challenges in data size, data types, and acquisition techniques limit the application of these standards to EM and XRM/XNH connectomics. To overcome these barriers, novel metadata standards must be developed while ensuring harmonization with existing standards for other modalities. To address this, the Big-Data Electron-Microscopy for Novel Community Hypotheses: Measuring And Retrieving Knowledge (BENCHMARK) team formed a working group to create image and experimental metadata standardization recommendations for the EM connectomics community. This document presents the BENCHMARK Image and Experimental Metadata Standards v1.1 for the connectomics community aiming to refine neuroimaging standards to support large-scale programs, archives, and tools. A companion paper describes standards for annotations and data products derived from these data.

## 2 Standards Development Process for Image and Experimental Metadata

In order to address community concerns about how to structure raw data and metadata for EM connectomics, we formed a community working group. This effort drew from numerous existing neuroscience communities that preserve, manage, and create metadata for different modalities. In the EM Connectomics community, three key areas for standardization were identified as:

- Archive formats for raw image files
- Imaging and Experimental Metadata
- Annotation data and metadata derived from image data

The working group was drawn from active members of the connectomics, EM, and neuroinformatics communities. Representation included students, staff, and investigators. Participants were drawn from the North American and European scientific communities. Universities, non-profit research centers, for-profit companies, and government organizations were represented. The Imaging Working Group contained experimentalists with extensive imaging experience, data scientists, and informatics experts with large scale processing and data storage experience for XRM and EM imaging modalities. Additional meetings were held with individual laboratories as well as small-group discussions on targeted topics.

This document addresses recommendations for 1) Archive formats for raw image files and 2) Imaging and Experimental Metadata standards. Considering the complexity of standards development and community adoption, we envision ongoing review by the community and working group, including open participation and contributions at www.github.com/aplbrain/benchmark-metadata. We encourage prototype implementations of these standards in EM community tools to enhance accessibility, interoperability, and reusability of data across various archives.

### 2.1 EM Connectomics Image and Experimental Metadata Standards Development Process

The working group met to discuss key guiding principles of standards development in this field, including:

- Building on existing file formats and standards efforts when possible
- Promoting inter-operability across EM Connectomics archives/tools
- Promoting accessibility and findability across modalities
- Planning for scale and distributed cloud-based computing

During working group meetings, subgroups were formed to discuss image and data file types, linking between datasets, potential multimodal experiments and cross-archive analysis. An emphasis was placed on preserving existing workflows, both within laboratories and existing software tools. Image formats and metadata standards were proposed to address issues during inter-operation (e.g. data ingest), data dissemination, and secondary data analysis. The working group recommended these standards be implemented in software tools for data archives and processing software to enable inter-operability. After each working group meeting, recommendations were aggregated by the core standards team. Draft documents are available at https://github.com/aplbrain/benchmark-metadata for continued community refinement.

### 2.2 Harmonization Efforts with Existing Standards and Datasets

**Figure 1:**
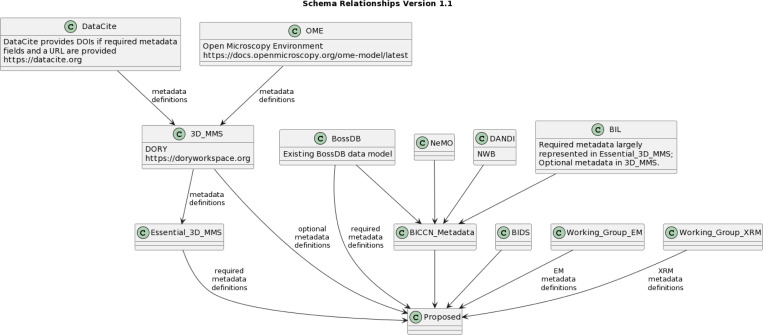
Overview of existing datasets, archives, and standards reviewed during the development process. This approach was critical to harmonization of the proposed standards with existing approaches to maximize reuse and promote interoperability.

In addition to working group meetings, extensive review of several existing, related standards and datasets was conducted (Fig 1). This was intended to leverage existing standards, harmonize efforts across archives, and promote a pathway to sustainability, possibly as an extension to an existing standards effort. Continued refinement of these draft standards will focus on harmonization with other metadata specifications. The reviewed standards included:

- Metadata services for the archives NEMO [25], DANDI [20], BossDB [13], DViD [17], webKnossos [30] and BIL [24]
- The standard for 3D Microscopy [36]
- Neurodata Without Borders Standard [20]
- Brain Imaging Data Structure (BIDS) Standard [35]
- Existing standards of the Volume EM community [37]

When possible, metadata fields utilize common terminology with the field. When conflicts exist, a harmonization mapping will be maintained by the BENCHMARK standards community. In addition, 38 extant datasets in the BossDB archive were reviewed. From these, it was determined what project metadata has already been generated. Moreover, a preliminary standardization was created for each dataset. This formed the basis of a prototype implementation of the BENCHMARK Standard (with programmatic RESTful API) at metadata.bossdb.org.

Below, we outline v1.1 of the BENCHMARK Image and Experimental Metadata Standards. We anticipate the community will continue to refine and develop this in the future. The metadata is developed to include information on raw image formats, experimental metadata, coordinate frames, image quality, and co-registration to datasets. The proposed metadata captures key aspects of the project: the title, contributors, species, date that the dataset was created, and other information that will be discussed throughout the paper. It also documents the format and structure of the metadata.

## 3 BENCHMARK EM Connectomics Standard v1.1

Based on Working Group input and meetings, we have formulated v1.1 BENCHMARK standards for Image Data and Experimental Metadata. We encourage adoption of this framework by the community, and continued revision to support emerging efforts, needs, and new technologies. These standards are recommended primarily for data dissemination to create FAIR datasets [18] (for example on archives, consortia portals, or project websites), rather than prescribing a particular approach to data manipulation within an experimental pipeline. Working versions of the standard are maintained at https://github.com/aplbrain/benchmark-metadata.

### 3.1 Raw Image Formats

EM and XRM connectomics datasets consist of high-resolution two-dimensional or three-dimensional neuroimaging datasets. Two-dimensional datasets are typically aligned to create a contiguous three-dimensional volume, which is the typical type of data volume stored in online archives. The field of connectomics therefore overlaps heavily with other three-dimensional neuroimaging domains but has particular aspects of data scale, unique proofreading workflows, and secondary data products which require consideration. We aim to leverage existing standards as much as possible, and aim to promote cross-archive and project compatibility. We recognize, however, that individual laboratories and software projects have significant investments in tools and formats and may need to maintain these approaches. However, adhering to the recommended standards on publication/export of the data products can greatly enhance interoperability and reuse in the community and further motivate generation and sharing of new datasets.

#### 3.1.1 General Desirable Properties of Image and Data Formats

A subgroup convened to identify key features for data formats to store three-dimensional neuroimaging datasets. Key factors were to create formats suitable for secondary analysis, and export between tools/archives. Key considerations identified were:

- Common, non-evolving formats
- Ability to create wrappers around existing tools/ecosystems
- Linkages between imagery data and other data types
- Fundamental concepts of data sharding [38] should be followed
- Consider both the individual laboratory and the needs of a community user
- Versioned releases likely sufficient for most secondary analysis

#### 3.1.2 Image Formatting Options

Promising existing formats were identified which have gained traction within the neuroimaging and biomedical imaging communities. It is recognized existing tools may have and may need to maintain internal data formats, but standard formats are required for interoperability. Version 1.1 of the BENCHMARK standards requires imaging data be stored in one of the following formats:

- OME-Zarr (version 2 of the Zarr specification) - to be updated to version 3 of the Zarr specification when appropriate [39]
- Cloud-volume [31]
- Neuroglancer precomputed [32]
- N5 [40]

These formats are broadly supported in the community, and tools such as TensorStore [41] can be utilized to provide a uniform API for reading and writing multiple array formats, including OME-Zarr, N5, and Neuroglancer precomputed. If a tool/archive uses an internal format, one or more of the above should be supported for compliance with the BENCHMARK v1.1 standard. Key issues to address moving forward include tracking provenance of data files, with immutable logs, versioned releases, and tracking of changes. Additional considerations include label mapping from data files to annotation IDs, an ongoing area of coordination between the raw image formats and annotation metadata formats. Additionally, support for lossless and lossy compression needs to be standardized.

### 3.2 Experimental Metadata for Projects

**Figure 2:**
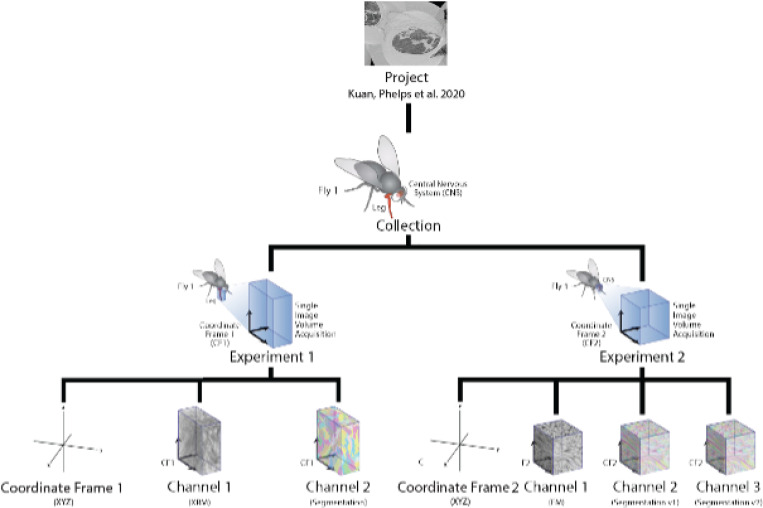
Simplified data model for storage of data, derived from a project hosted at BossDB (https://bossdb.org/project/kuan_phelps2020). This uses an example dataset including samples from Drosophila in multiple coordinate frames [42] The full metadata structure can be seen at https://github.com/aplbrain/benchmark-metadata

**Figure 3:**
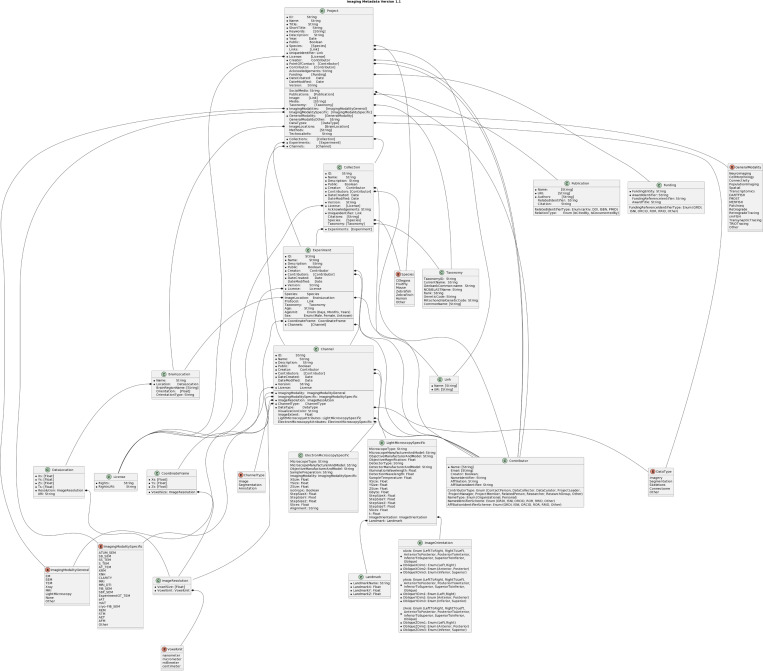
Overview of existing datasets, archives, and standards reviewed during the development process. This approach was critical to harmonization of the proposed standards with existing approaches to maximize reuse and promote interoperability.

Fields documented in imaging and metadata standard have 18 classes that are used to denote metadata for a given project. This includes the structure of data channels and experiments within a project (Fig. 2). The metadata fields allow values consisting of strings, integers, enumerations, floats or Booleans, and are essential elements to help define the projects fully. The classes provide fields that gives the field name, description of the field name, and allowed values. The metadata encompasses titles of projects, names of contributors, creators, URIs, and more (Table 1). This information can be vital to have when it comes to type of searches and queries, such as project types, specimens’ ages, sex, organizations involved with a particular project, and more. The full relationship of all classes, fields, and enumerations are shown in Fig. 3.

#### 3.2.1 Project

The projects class contains 36 fields that give a high-level description of a dataset. These high-level descriptions include the title, short title, description, year, the DOI (Digital Object Identifier), and more (Table 1). The project entity is the highest-level object which gives an overview of the data available in a dataset. This enables users to reference a list of projects with a thorough description and query key project metadata. String identifiers allow for easy search, and embedded identifiers ensure data satisfies FAIR principles. Boolean values represent if a project is public or has a publication, and each project links to related papers and indicates any modification to the project. The project contains links to the collections which make up the project.

#### 3.2.2 Collection

The collection class consists of 18 fields used to associate a set of experiments within a project. A project may contain multiple collections. Collections may associate a set of experiments from a single topic or from a single publication. These may have separate creators, identifiers, and licenses from a project, so long as they are a subset of the overall project creators, identifiers, and licenses. A collection may contain experiments with different coordinate frames (e.g. different brain regions).

#### 3.2.3 Experiment

The experiments class consists of 20 fields defining a set of data which share a common coordinate frame. An experiment consists of one or more Channels associated with the coordinate frame. An experiment may have separate creators, identifiers, and licenses from a collection, as long as they are a subset of the overall collection creators, identifiers, and licenses. It also includes a field for an identifier for an experimental protocol. An experiment may contain channels with different data types, such as raw imagery and segmentations.

#### 3.2.4 Channel

The channel class consists of 19 fields defining a three-dimensional volume of imaging data, with a defined coordinate frame. The data may consist of different types, such as unsigned integer or float, but a given channel may only have one type of data. A channel has a uniform resolution for each of the X, Y, and Z dimensions (which may differ between them). A channel may have creators, licenses, and identifiers which are separate from the experiment, as long as they are a subset of the overall experiment creators, identifiers, and licenses. A channel has one associated coordinate frame (which must be the same as the experiment coordinate frame).

#### 3.2.5 CoordinateFrame

The CoordinateFrame class consists of four entities specifying an extent for the X, Y, and Z axis of a three-dimensional volume. Each dimension is specified as a minimum and maximum float value in a list. The resolution of each voxel (in X, Y and Z) is specified for the CoordinateFrame in the VoxelSize field. This is an ImageResolution type which specifies the x,y, and z resolution along with the relevant unit. It is recommended, but not required, that CoordinateFrame objects share a common reference within a project. Future work will consider mappings to common coordinate frameworks, as they are established in the field.

### 3.3 Experimental Metadata Classes

#### 3.3.1 BrainLocation

The BrainLocation class contains 4 fields to capture the location of a coordinate frame within a larger brain volume. This contains information about the spatial position and orientation of a coordinate frame within a brain. It also contains a string name of the region. If this class exists for an experiment, it is recommended, but not required, that the information is consistent with an established coordinate frame.

#### 3.3.2 DataLocation

The DataLocation class contains 5 fields which capture the spatial extent and origin point of a volume within a coordinate frame. It also captures the image resolution in each dimension.

#### 3.3.3 LightMicroscopySpecific

This class is derived from the Standard metadata for 3D microscopy [36] and is included to promote compatibility with this standard. Future work will investigate further methods for co-registration and harmonization of these standards. The LightMicroscopySpecific class contains 28 fields to capture the properties of channels and experiments imaged with light microscopy techniques. This includes details on the microscope as well as experimental settings.

#### 3.3.4 ElectronMicroscopySpecific

The ElectronMicroscopySpecific class consists of 14 fields which define metadata related to electron microscopes utilized to generate connectivity datasets. This includes information about the microscope itself as well as imaging techniques and settings.

#### 3.3.5 Taxonomy

The taxonomy class contains 8 fields that provide information to categorize and classify biological samples and species. These classes are Taxonomy ID, current name, GenBank common name, NCBI BLAST name, rank, genetic, mitochondrial genetic code, and common name. Taxonomy information can be found on the National Library of Medicine Taxonomy website [43].

#### 3.3.6 ImageOrientation

This class is derived from the Standard metadata for 3D microscopy [36], and is used to capture information related to image orientation angles in LightMicroscopySpecific.

#### 3.3.7 Landmark

This class is derived from the Standard metadata for 3D microscopy [36], and is used to capture information related to anatomical landmarks in LightMicroscopySpecific.

#### 3.3.8 ImageResolution

This class captures the resolution of the voxels in the volume. This is assumed to be uniform throughout the dataset, but is not assumed to be isotropic. Units for the resolution are also specified.

### 3.4 Project Metadata Classes

#### 3.4.1 Contributor

The contributor class consists of 11 fields that provide information about personnel associated with a specific project. This class gives information on the individual or group members who provided skills, knowledge, and resources for a particular project. The standard specifies the role that the individuals played towards the project, and each project is assigned a point of contact. There is also a minimum of one creator for each project. Out of the 11 fields, 6 fields in the contributor class are associated with digital identifiers. These identifiers include ORCID (Open Researcher and Contributor Identifier), RRID (Research Resource Identifiers), ISNI (International Standard Name Identifier), ROR (Research Organization Registry), and a few others (Table 1).

#### 3.4.2 License

Within the license class, there are 4 fields that give information about the licenses which govern usage of the data for this project. This specifies the terms and agreements that protect the rights of a project. In the license class (Table 1), the fields that are required include the URI to the rights, identifier, and corresponding DOI. The project may be associated with multiple licenses, which reference individual datasets within the given project. For example, all public projects on the archive BossDB are currently licensed under the Creative Commons Attribution 4.0 International License [44].

#### 3.4.3 Funding

The funding class contains 5 fields explaining the organization(s) that financially support a given project. The funding class will provide information regarding what corporation or grant provided resources to support the project. These resources can come from various sources (e.g., government, individual donors, etc.), and the fields that are included in the funders class are funding entity, award identifier, funding reference identifiers, funding resources identifiers type, and award title.

#### 3.4.4 Publication

In the publication class, there are 8 fields capturing information about a dataset, papers, and where publications can be found. This allows for tracking the impact of the work with a particular dataset. The fields that are included in the publication class are URI, authors, related identifiers, PubMed Central Identification (PMCID), relation type, and citation. This class is critical to give creditability and validity to the team which generated the dataset.

#### 3.4.5 Link

Within the link class, there are two fields. These field names are name and Uniform Resource Identifier (URI), and are used to capture external references and resources.

### 3.5 Enumerations

Several enumerations are defined for restricted fields in the standard. These include

- Species – common names of species used in a project, intended for human-interpretable search
- DataType – Data products included in a channel, such as segmentations or raw data
- ChannelType – Classifies a channel as raw data, a segmentation, or an annotation channel
- GeneralModality – Defines the general experimental modality of the dataset
- ImagingModalityGeneral – Defines the general imaging techniques used in the dataset
- ImagingModalitySpecific – Defines specific imaging types used in the dataset (e.g. FIB-SEM)
- VoxelUnit – Defines resolution units

## 4 Discussion

The BENCHMARK Image and Experimental Metadata Standards represent a preliminary consensus, drawing from our working group and existing standards efforts, to establish a robust framework for EM and XRM/XNH connectomics. The unique challenges inherent to EM data, such as immense size and diverse acquisition methods, demand specialized standards to ensure data integrity and interoperability. These standards address raw image formats, experimental metadata, coordinate frames, and publication details and underscores the commitment to comprehensive data management. These standards not only facilitate adherence to the FAIR principles but also set the stage for more streamlined collaboration and data sharing within the EM connectomics community.

The proposed standards build on and incorporate existing standards efforts in the community, including existing BRAIN Initiative Archives [20, 13, 24, 25], deployed solutions from the EM connectomics community [17, 31, 32, 33, 14, 16], and existing standards efforts [20, 36, 35]. The incorporation of existing file formats and standards in the BENCHMARK recommendations demonstrates a pragmatic approach, acknowledging the importance of building upon established practices. This strategy not only promotes interoperability among various EM Connectomics archives and tools but also facilitate the seamless integration of these standards into the existing research infrastructure. By prioritizing compatibility, the BENCHMARK standards aim to reduce barriers to adoption, ensuring a smooth transition for researchers while enhancing the overall accessibility and utility of EM connectomics data. This will be particularly critical to enabling multi-modal analysis [28] and secondary comparative connectomics analysis [45].

In the context of data scalability, the BENCHMARK standards exhibit a forward-looking perspective by emphasizing compatibility with cloud-based computing. For example, a preliminary prototype implementation is available (metadata.bossdb.org) which allows programmatic access of project metadata. This service is directly usable by individuals, but also allows for cross-archive indexing, creating real-time dashboards, and contributing to scientific gateways of large consortium projects.

We encourage continued evolution of these standards with input from the community, developers of tools, and emerging scientific consortia. Further work will be required to fully capture novel and unique experimental and imaging approaches, track software tools and methods used to process the data, and support emerging comparative experiments. Open community comment, discussion, and contributions are encouraged at the BENCHMARK standards community repository (www.github.com/aplbrain/benchmark-metadata). Future working group meetings will continue to oversee governance and development of new versions.

While evolution of these approaches is expected, this version of the BENCHMARK Image and Experimental Metadata Standards aims to provide a consistent set of image and experimental metadata information for the unique and emerging fields of EM and XRM connectomics. By addressing key aspects of data management and compatibility these standards contribute to the advancement of secondary analysis as well as cross-modality analysis. In particular, we hope these efforts will support emerging large-scale scientific efforts such as the NIH BRAIN CONNECTS program.

## Figures and Tables

**Table 1:** An overview of key metadata classes required for the BENCHMARK standards. It details various fields such as C Contributors, License, Funder, Projects, Taxonomy, Coordinate Frame, Publication, and Link, each with specific data types and requirements. This table serves as a reference for understanding the structure of metadata in connectomics, aiding in the organization, retrieval, and analysis of neuroscience data.

Field Name	Description	Data Type	Required

Contributor			
Name	Individuals, organizations, or entities who contributed to or are responsible for a project	[String]	Yes
Email	Contact email	[String]	No
Creator	Identifies contributor who created a dataset	Boolean	No
ContributorType	Categorization of the role of the contributor. Recommended: ProjectLeader, ResearchGroup	Enum (ContactPerson, DataCollector, DataCurator, ProjectLeader, ProjectManager, ProjectMember, RelatedPerson, Researcher, ResearchGroup, Other)	No
NameType	Type of contributor	Enum (Organizational, Personal)	No
NameIdentifier	Identifier of individual or entity that created contribution	String	No
NameIdentifier Scheme	Identifying scheme used in NameIdentifier	Enum (GRDI, ISNI, ORCID, ROR, RRID, Other)	No
Affiliation	Organization associated with individual contributor a particular project	String	No
AffiliationIdentifier	Affiliation Identifiers are unique values assigned to affiliation	String	No
AffiliationIdentifier Scheme	Identifying scheme used in AffiliationIdentifier	Enum (GRDI, ISNI, ORCID, ROR, RRID, Other)	No

License			

Rights	License which defines usage of the data	String	Yes
RightsURI	The rightsURI is a digital resource providing information about the license of a project	String	Yes

Funding			

FundingEntity	The individual or organization who are providing financial support to a project	String	Yes
AwardIdentifier	Award identifier provides an award or grant number or name	String	Yes
FundingReference Identifier	Identifier for the funding source	String	No
FundingReference IdentifierType	Type of funding source identifier	Enum (GRDI, ISNI, ORCID, ROR, RRID, Other)	No
AwardTitle	The name of the award or grant from funding entity	String	No

Projects			

Title	Title for a specific project	String	Yes
ShortTitle	Display title of less than 100 characters	String	Yes
ID	Provides short, unique identifier for project	String	Yes
Keywords	Keyword descriptors for the project	[String]	Yes
Description	Provides information of species type, image modality, and brief definition of the project	String	Yes
Public	Indicates whether the dataset is public (true) or restricted (false)	Boolean	Yes
Year	Year project was created	Date	Yes
Publications	Publications linked to project	[Publication]	No
Links	External links associated with project	[Link]	No
License	One or more licenses associated with the use of this project	[License]	Yes
Creator	Individual who created this project	Contributor	Yes
PointOfContact	Point of contact for this project, may be multiple people	[Contributors]	Yes
Contributor	Persons who contributed to creation of this project	[Contributor]	Yes
Acknowledgements	Acknowledgements statement	String	No
Funding	Information about funding agencies	[Funding]	No
Species	Organism type	[Species]	Yes
Taxonomy	Taxonomy of organisms in project	[Taxonomy]	No
DateCreated	Gives the date for when the project was created	Date	Yes
Channels	Channels associated with this project. Must be at least one	[Channel]	Yes
Experiments	Experiments associated with this project. Must be at least one	[Experiments]	Yes
Collections	Collections associated with this project. Must be at least one	[Collection]	Yes
Media	Associated media information or links	[String]	No
DataTypes	Types of data contained in experiments associated with this project	[DataType]	No
Image	Files or links of images associated with this project, for display	[Link]	No
TechnicalInfo	Additional technical information	String	No
Methods	Identifiers linking to description of methods (paper or protocols.io, for instance)	[String]	No
SocialMedia	Website, social media account, or description associated with project	String	No
GeneralModality	Gives list of general techniques and approaches associated with this dataset. This may indicate additional modalities associated with the project, beyond microscopy data	[GeneralModality]	Yes
GeneralModalityOther	Specification of other modalities not described in standard	String	No
ImagingModalities	Gives a list of modalities contained in this project	[ImagingModality General]	Yes
ImagingModality Specific	Specific imaging types used in this project	[ImagingModality Specific]	No
ImageLocations	Locations of experimental volumes within the brain	[BrainLocation]	No
Version	Current version of project	String	Yes
UniqueIdentifier	Unique identifier for dataset (DOI preferred)	Link	No
DateModified	Gives the date for when the project was updated or changed	Date	No

Taxonomy			

TaxonomyID	A unique numerical identifier that is assigned to a specific organism in the NCBI Taxonomy database	Int	Yes
CurrentName	The organism type in its scientific form. Ex: Mus musculus, Drosophila, C. elegans	String	No
GenBankCommonName	Provides a simplified and recognizable name for a specific organism	Integer	No
NCBIBlastName	Blast name provided by NCBI	String	No
Rank	Categorize and organize organisms based on their evolutionary relationships. Ex. Species, genus, family, etc	String	No
GeneticCode	Genetic information regarding the species	String	No
Mitochondrial GeneticCode	Mitochondrial genetic information regarding the species.	String	No
CommonName	The everyday name of a species, organism, or biological entity	[String]	No

CoordinateFrame			

Xs	Numerical value represented horizontally in two-dimensions	[Float]	Yes
Ys	Numerical value represented vertically in two-dimension	[Float]	Yes
Zs	Numerical value representing height in three-dimension	[Float]	Yes
VoxelSize	The resolution of each 3D voxel	ImageResolution	Yes

Publication			

Name	Name of publication	[String]	Yes
URI	Uniform Resource Identifier for affiliated web resource	[String]	Yes
Authors	The creators or writers of the work	[String]	Yes
RelatedIdentifier	Identifier for publications.	String	No
RelatedIdentifierType	Type of publication identifier.	Enum (arXiv, DOI, ISBN, PMID, Other)	No
RelationType	Relationship to publication.	Enum (IsCitedBy, IsDocumentedBy)	No
Citation	Preferred citation for this publication.	String	No

Link			

Name	Provides the name of website or resource	[String]	Yes
URI	Uniform Resource Identifier pathway to the website or resource	[String]	Yes

**Table 2:** This chart serves as a reference of commonly used acronyms for the fields used in the BENCHMARK standards.

Abbreviation Techniques	Description

AET	Analytical Electron Microscopy
AFM	Atomic Force Microscopy
AT_TEM	Array Tomography Transmission Electron Microscope
ATUM_SEM	Automated Tape Collecting Ultramicrotome Scanning Electron Microscope
CLARITY	Clear Lipid-exchanged Acrylamide-hybridized Rigid Imaging / Immunostaining / in situ-hybridization-compatible Tissue hYdrogel
Cryo_FIB_SEM	Cryogenic focused Ion Beam-Scanning Electron Microscope
FIB_SEM	Focused Ion Beam-Scanning Electron microscope
LEEM	Low-Energy Electron Microscopy
LM	Light Microscopy
PEEM	Photoemission Electron Microscopy
mAT	Magneto Acoustic Tomography
MRI	Magnetic Resonance Imaging
REM	Reflection Electron Microscope
sAT	Scanning Acoustic Tomography
SB_SEM	Serial Block-Face Scanning Electron Microscope
SEM	Scanning Electron Microscope
S_TEM	Scanning Transmission Electron Microscopy
SS_TEM	Serial Section Transmission Electron Microscopy
STM	Scanning Tunneling Microscope
TEM	Transmission Electron Microscope
XRM	Xray Microscopy

Identifiers	

GRID	Global Research Identifier Database
ISNI	International Standard Name Identifier
ORCID	Open Researcher and Contributor ID
PMCID	PubMed Central Identification
ROR	Research Organization Registry
RRID	Research Resource Identifier
URI	Uniform Resource Identifier

Other	

NCBI	National Center for Biotechnology Information
